# Supplementary material for: Probabilistic program inference in network-based epidemiological simulations
Source: PLoS Comput Biol. 2022 Nov 7;18(11):e1010591. doi: 10.1371/journal.pcbi.1010591 (PMC9671460; doi:10.1371/journal.pcbi.1010591)
Supplement: S1 Appendix — We restate the derivation of the technique of blackbox variational inference, including the ELBO objective and the score function ELBO gradient. (PDF) [file pcbi.1010591.s001.pdf]

S1 Appendix. Blackbox Variational Inference

We explain the derivation for Blackbox Variational Inference (BBVI, [1], [2]). In the following discussion, we use x to refer to observed data (i.e. regional infection statistics), and z to refer to the set of all latent variables (such as the initial community exposure rates $\rho_{1:C}$ and the exposure probabilities $\beta^E_{1:t_N}, \beta^I_{1:t_N}$). We use Stochastic Variational Inference (SVI) to approximate the intractable posterior $p(z|x)$ by optimizing the parameters of a variational distribution $q(z; \phi)$ (also denoted q_ϕ) from which we are able to directly draw samples.

ELBO Objective. A common approach to optimizing the variational distribution is to find parameters ϕ^* that minimize the exclusive Kullback-Leibler (KL) divergence between the variational approximation and the posterior:
$\phi^* = \arg\min_\phi \mathrm{KL}(q_\phi(z) \| p(z \mid x))$,

$$\begin{aligned}
\mathrm{KL}(q_\phi || p) &= \mathbb{E}_{z \sim q_\phi}\left[\log \frac{q_\phi(z)}{p(z|x)}\right] &(1)\\
&= \mathbb{E}_{z \sim q_\phi}\left[\log \frac{q_\phi(z)}{p(x,z)} + \log p(x)\right] &(2)\\
&= \mathbb{E}_{z \sim q_\phi}\left[\log \frac{q_\phi(z)}{p(x,z)}\right] + \log p(x). &(3)
\end{aligned}$$

Minimizing this exclusive KL divergence is equivalent to maximizing a lower bound on the log marginal likelihood of the data. Since the marginal likelihood $p(x)$ is also referred to as the "evidence", this bound is called the Evidence Lower BOund (ELBO),

$$\mathcal{L}_\phi = \underbrace{\mathbb{E}_{z \sim q_\phi}\left[\log \frac{p(x,z)}{q_\phi(z)}\right]}_{\text{ELBO}} = \log p(x) - \underbrace{\mathrm{KL}(q_\phi(z)||p(z|x))}_{\geq 0} \leq \underbrace{\log p(x)}_{\text{log evidence}} . \qquad (4)$$

Score Function ELBO Gradient. Variational Autoencoders [3, 4] and related methods optimize the ELBO by computing reparametrized gradient estimates, which require the generative model $p(x, z)$ to be differentiable with respect to the latent variables z. These reparametrized gradient estimators have the advantage of low variance (meaning that relatively little sampling and computation is required per gradient step), but impose limits on the generative models being studied. Specifically, models that incorporate discrete variables and control flow will not be differentiable, and it may be infeasible or undesirable to find a continuous approximation to make such a model differentiable. We therefore choose to maximize the ELBO using a so-called "score-function" gradient estimator that does not require reparameterization. This approach is generally referred to as Blackbox Variational Inference (BBVI) [1, 2] We repeat the derivation of this gradient estimator here for convenience,

$$\nabla_\phi \mathcal{L}_\phi = \nabla_\phi \mathbb{E}_{z\sim q_\phi}\left[\log \frac{p(x,z)}{q_\phi(z)}\right] \tag{5}$$

$$= \int dz\ \nabla_\phi \left(q_\phi(z)\log\frac{p(x,z)}{q_\phi(z)}\right) \tag{6}$$

$$= \int dz\ \nabla_\phi q_\phi(z)\log\frac{p(x,z)}{q_\phi(z)} + q_\phi(z)\nabla_\phi \log\frac{p(x,z)}{q_\phi(z)} \tag{7}$$

$$= \int dz\ q_\phi(z)\log\frac{p(x,z)}{q_\phi(z)}\nabla_\phi \log q_\phi(z) + q_\phi(z)\nabla_\phi \log\frac{p(x,z)}{q_\phi(z)} \tag{8}$$

$$= \int dz\ q_\phi(z)\log\frac{p(x,z)}{q_\phi(z)}\nabla_\phi \log q_\phi(z) - q_\phi(z)\nabla_\phi \log q_\phi(z) \tag{9}$$

$$= \mathbb{E}_{z\sim q_\phi}\left[\left(\log\frac{p(x,z)}{q_\phi(z)} - 1\right)\nabla_\phi \log q_\phi(z)\right] \tag{10}$$

$$= \mathbb{E}_{z\sim q_\phi}\left[\left(\log\frac{p(x,z)}{q_\phi(z)}\right)\nabla_\phi \log q_\phi(z)\right] - \underbrace{\mathbb{E}_{z\sim q_\phi}\left[\nabla_\phi \log q_\phi(z)\right]}_{=0} \tag{11}$$

$$\approx \frac{1}{K}\sum_{k=1}^{K}\left(\log\frac{p(x,z^k)}{q_\phi(z^k)}\right)\nabla_\phi \log q_\phi(z^k), \qquad z^k \sim q_\phi(z). \tag{12}$$

The above gradient estimator does not require the generative model to be differentiable; we only need to sample from the variational distribution and evaluate both the generative model and variational models pointwise. While score function gradient estimators are known to have higher variance and require smaller step sizes than other approaches, they are unbiased estimates of the gradient, and hence the corresponding stochastic gradient descent updates will converge under the usual Robbins and Monro conditions for stochastic approximation algorithms [5].

To reduce the variance of this estimator various techniques have been proposed, including the introduction of control variates [6, 7, 8, 9], which aim to reduces the variance of the estimator while not changing the expectation of the gradient estimator. A common choice is to use a constant baseline b, which can easily be shown to leave the expectation above invariant,

$$\nabla_\phi \mathcal{L}_\phi = \mathbb{E}_{z\sim q_\phi}\left[\left(\log\frac{p(x,z)}{q_\phi(z)}\right)\nabla_\phi \log q_\phi(z)\right] - b\,\underbrace{\mathbb{E}_{z\sim q_\phi}\left[\nabla_\phi \log q_\phi(z)\right]}_{=0} \tag{13}$$

$$\approx \frac{1}{K}\sum_{k=1}^{K}\left(\log\frac{p(x,z^k)}{q_\phi(z^k)} - b\right)\nabla_\phi \log q_\phi(z^k), \qquad z^k \sim q_\phi(z). \tag{14}$$

Notably, an optimal baseline, in terms of the variance of the estimator, can be derived analytically, but requires estimating additional covariance terms. Hence, in practice, the expected log-weight b can simply be estimated as a sample average $\hat{b}$,

$$b = \mathbb{E}_{z\sim q_\phi}\left[\log\frac{p(x,z)}{q_\phi(z)}\right] \approx \hat{b} = \frac{1}{K}\sum_{k=1}^{K}\log\frac{p(x,z^k)}{q_\phi(z^k)}, \qquad z^k \sim q_\phi(z). \tag{15}$$

This results in a baseline which is computationally efficient and easy to implement. While it is not guaranteed to reduce the variance and introduces a small bias for finite sample sizes, it is a standard choice and tends to works well in practice (see [10] for a review).

References

1. Wingate D, Weber T. Automated Variational Inference in Probabilistic Programming. arXiv preprint arXiv:13011299. 2013;.

2. Ranganath R, Gerrish S, Blei D. Black Box Variational Inference. In: Artificial Intelligence and Statistics. PMLR; 2014. p. 814–822.

3. Kingma DP, Welling M. Auto-Encoding Variational Bayes. International Conference on Learning Representations. 2013;.

4. Rezende DJ, Mohamed S, Wierstra D. Stochastic Backpropagation and Approximate Inference in Deep Generative Models. In: International Conference on Machine Learning. PMLR; 2014. p. 1278–1286.

5. Robbins H, Monro S. A Stochastic Approximation Method. The Annals of Mathematical Statistics. 1951; p. 400–407.

6. Weber T, Heess N, Buesing L, Silver D. Credit Assignment Techniques in Stochastic Computation Graphs. arXiv:190101761 [cs, stat]. 2019;.

7. Mnih A, Rezende D. Variational Inference for Monte Carlo Objectives. In: International Conference on Machine Learning; 2016. p. 2188–2196.

8. Tucker G, Mnih A, Maddison CJ, Lawson J, Sohl-Dickstein J. REBAR: Low-Variance, Unbiased Gradient Estimates for Discrete Latent Variable Models. In: Advances in Neural Information Processing Systems; 2017. p. 2624–2633.

9. Grathwohl W, Choi D, Wu Y, Roeder G, Duvenaud D. Backpropagation through the Void: Optimizing Control Variates for Black-Box Gradient Estimation. International Conference on Learning Representations. 2018;.

10. Mohamed S, Rosca M, Figurnov M, Mnih A. Monte Carlo Gradient Estimation in Machine Learning. J Mach Learn Res. 2020;21(132):1–62.
