# Supplementary material for: Probabilistic program inference in network-based epidemiological simulations
Source: PLoS Comput Biol. 2022 Nov 7;18(11):e1010591. doi: 10.1371/journal.pcbi.1010591 (PMC9671460; doi:10.1371/journal.pcbi.1010591)
Supplement: S2 Appendix — We explain the details of the Analytic Rt-matched parameter estimation and Certainty-Equivalent Expectation Maximization baseline methods. (PDF) [file pcbi.1010591.s002.pdf]

S2 Appendix. Baseline Methods

We compare our probabilistic programming-based parameter inference method to several more traditional methods using compartmental disease models with simplifying assumptions. Note that our method fits the parameters of a disease simulator to past data, but does not model evolving temporal dynamics. Thus we compare against other methods of parameter fitting and not against forecasting methods such as [1].

Analytic R_t-matched Parameters. We derive constant f_{NSEIR} parameters β^E, β^I per edge, so that each lead to COVID-19 R_t values reported in literature. We compute an effective reproductive number R_t as,

$$R_t(\beta^E, \gamma) = \mathbb{E}_{u \in \mathcal{V}} \left[\sum_{v \in N(v)} \frac{W_{uv}\beta^E}{1 - (1 - W_{uv}\beta^E)(1-\gamma)} \right]. \quad (1)$$

COVID-19 R_t values across the world have been reported to be roughly between 1.4 and 5 [2, 3]. These values reflect primarily asymptomatic transmission, therefore a value of R_t in this range is suitable for the estimation of β^E. For our calculations we set $R_t = 3$ and $\gamma = 0.048$. We approximate the expectation over neighbors by considering the top 25% highest degree nodes. Note that this solution implicitly assumes uniform edge probabilities and constant average degree, a deviation from our network generation modeling choices, where we explicitly model community structured interactions and heterogeneous degree distribution.

Note that β^E and β^I cannot be simultaneously identified from R_t alone. To estimate β^I, we again apply (1) by solving $R_t(\beta^I, \gamma) = 0.25$ for β^I. For β^I, we use an R_t of 1/12 the size, since this ratio holds for the R_t values computed from the compartmental model fit to data using CE-EM. Specifically, we found the expected number of exposures caused by each infected individual is 1/12 the number caused by each exposed individual.

Since the R_t-Analytic method provides only a point estimate of parameters, to fairly compare this method to our method as in Table 2 we set the variance of our variational posterior q_ϕ to 0.

CE-EM: Certainty-Equivalent Expectation Maximization
Certainty-Equivalent Expectation Maximization (CE-EM) [4, 5] is algorithm for system identification for time series models. It assumes that there exists only one state trajectory $x_{1:T}$ that satisfies the system dynamics and is consistent with the observed data $y_{1:T}$, i.e.,

$$p(x_{1:T} \mid y_{1:T}, \theta) = \delta_{x^{\text{ML}}_{1:T}(\theta)}(x_{1:T}), \qquad x^{\text{ML}}_{1:T}(\theta) = \underset{x_{1:T}}{\arg\max}\, p(x_{1:T} \mid y_{1:T}, \theta). \quad (2)$$

These assumptions allow for a simplified EM-algorithm, which alternately computes updates to the state trajectory $x_{1:T}$) and the system parameters θ. Beginning with the current estimated parameters $\theta^{(k)}$,

$$\begin{aligned} x^{(k+1)}_{1:T} &= \underset{x_{1:T}}{\arg\max}\, p(x_{1:T} \mid y_{1:T}, \theta^{(k)}) = \underset{x_{1:T}}{\arg\max}\, p(x_{1:T}, y_{1:T} \mid \theta^{(k)}), \\ \theta^{(k+1)} &= \underset{\theta}{\arg\max} \int dx_{1:T}\ \log p(x_{1:T}, y_{1:T} \mid \theta)\delta_{x^{(k+1)}_{1:T}}(x_{1:T}) \\ &= \underset{\theta}{\arg\max}\ \log p\left(x^{(k+1)}_{1:T}(\theta_k), y_{1:T} \mid \theta\right). \end{aligned}$$

In practice, due to its rather restrictive assumptions, CE-EM is best suited for time series models with nearly deterministic system evolution and uni-modal posterior distribution $p(x_{1:T} \mid y_{1:T})$. We use Certainty-Equivalent Expectation Maximization (CE-EM) as method for fitting the disease parameters of the differentiable compartmental SEIR [6].

In our application, we consider $\theta = (\beta^E, \beta^I, \gamma, \lambda_R, \lambda_D)$ a set of disease parameters. Mean counts $\mu_{x_{1:T}} = (S_{1:T}, E_{1:T}, I_{1:T}, R_{1:T}, D_{1:T})$ can be computed from the SEIR differential equations using numerical integration. The probability can be factored as,

$$\begin{aligned} p\left(x_{1:T}, y_{1:T} \mid \theta\right) &= p\left(x_{1:T} \mid \theta\right) p\left(y_{1:T} \mid x_{1:T}, \theta\right) \\ &= p\left(x_1 \mid \theta\right) \prod_i p\left(x_i \mid x_{i-1}, \theta\right) \prod_i p\left(y_i \mid x_i, \theta\right). \end{aligned}$$

These probabilities are given by a Gaussian noise model defined by $\mu_{x_{1:T}}$ and some fixed noise parameters and can be computed analytically.

References

1. CDC. Forecasts of COVID-19 Deaths; 2022. Available from: `https://www.cdc.gov/coronavirus/2019-ncov/science/forecasting/forecasting-us.html?CDC_AA_refVal=https%3A%2F%2Fwww.cdc.gov%2Fcoronavirus%2F2019-ncov%2Fcovid-data%2Fforecasting-us.html`.

2. Adam D. A Guide to R—the Pandemic's Misunderstood Metric. Nature. 2020;583(7816):346–348.

3. Li Q, Guan X, Wu P, Wang X, Zhou L, Tong Y, et al. Early Transmission Dynamics in Wuhan, China, of Novel Coronavirus-infected Pneumonia. New England Journal of Medicine. 2020;382(13). doi:10.1056/NEJMoa2001316.

4. Goodwin GC, Aguero JC. Approximate EM algorithms for Parameter and State Estimation in Nonlinear Stochastic Models. In: Proceedings of the 44th IEEE Conference on Decision and Control. IEEE; 2005. p. 368–373.

5. Menda K, De Becdelievre J, Gupta J, Kroo I, Kochenderfer M, Manchester Z. Scalable Identification of Partially Observed Systems with Certainty-Equivalent EM. In: International Conference on Machine Learning. PMLR; 2020. p. 6830–6840.

6. Menda KR, Laird L, Kochenderfer MJ, Caceres RS. Explaining COVID-19 Outbreaks with Reactive SEIRD Models. medRxiv. 2021;.
