# Supplementary material for: Probabilistic program inference in network-based epidemiological simulations
Source: PLoS Comput Biol. 2022 Nov 7;18(11):e1010591. doi: 10.1371/journal.pcbi.1010591 (PMC9671460; doi:10.1371/journal.pcbi.1010591)
Supplement: S3 Appendix — We explain our hyperparameter selection. We describe how the initial exposed fraction was handled for the baseline Rt analytic and CE-EM methods. Finally, we show results of the CE-EM baseline method. (PDF) [file pcbi.1010591.s003.pdf]

S3 Appendix. Additional Experimental Details and Results

Hyperparameter Tuning We list the hyperparameter values we tried in Table A. We selected the bold parameters based on training stability and goodness of fit.

Hyperparameter	Range
Observational Noise Scaling (ν)	0.00025, 0.00035, **0.0005**
Learning Rate	10^{-6} ,$\mathbf{10^{-5}}$,$5 \cdot 10^{-4}$, 10^{-4}
Samples Per Iteration	20,50,75,**120**, 200
Prior Initial Exposed Logit Mean (μ_c^ρ)	$-\mathbf{7}$, -6.5, -6
Prior Initial Exposed Logit StdDev (σ_ρ)	**0.0**, 1.0, 1.6
Prior β^E Logit Mean (μ_{β^E})	-2.2, $-\mathbf{1.39}$, -0.85
Prior shared disease parameter StdDev ($\sigma_{\beta^E}, \sigma_{\beta^I}, \sigma_\lambda, \sigma_\gamma$)	**0.0**, 0.47, 1.16
Expected Initial Exposed Fraction E_0	0.02, **0.04**, 0.08

Table A. Hyperparameter tuning ranges. Bold values correspond to best performance.

Setting Initial Exposed for Baselines Both the compartmental SEIR model and topological-SEIR model require an initial exposure percentage. However, neither the CE-EM nor the R_t-analytic method fits this value. Thus to compare our models fairly, we have used the best MDAE over six different initial exposures: 1) uniform exposure of 0.5%, 2) uniform exposure of 1%, 3) uniform exposure of 2%, 4) uniform exposure of %5, 5) inferred initial exposure using BBVI with high observational noise $\nu = 0.0005$, 6) inferred initial exposure using BBVI with low observational noise $\nu = 0.00025$.

Baseline CE-EM results See S1 Fig for plots of daily SEIR and cumulative infection counts simulated using SEIR equations using parameters estimated by CE-EM.
