# Supplementary figures and images for: Probabilistic program inference in network-based epidemiological simulations
Source: PLoS Comput Biol. 2022 Nov 7;18(11):e1010591. doi: 10.1371/journal.pcbi.1010591 (PMC9671460; doi:10.1371/journal.pcbi.1010591)

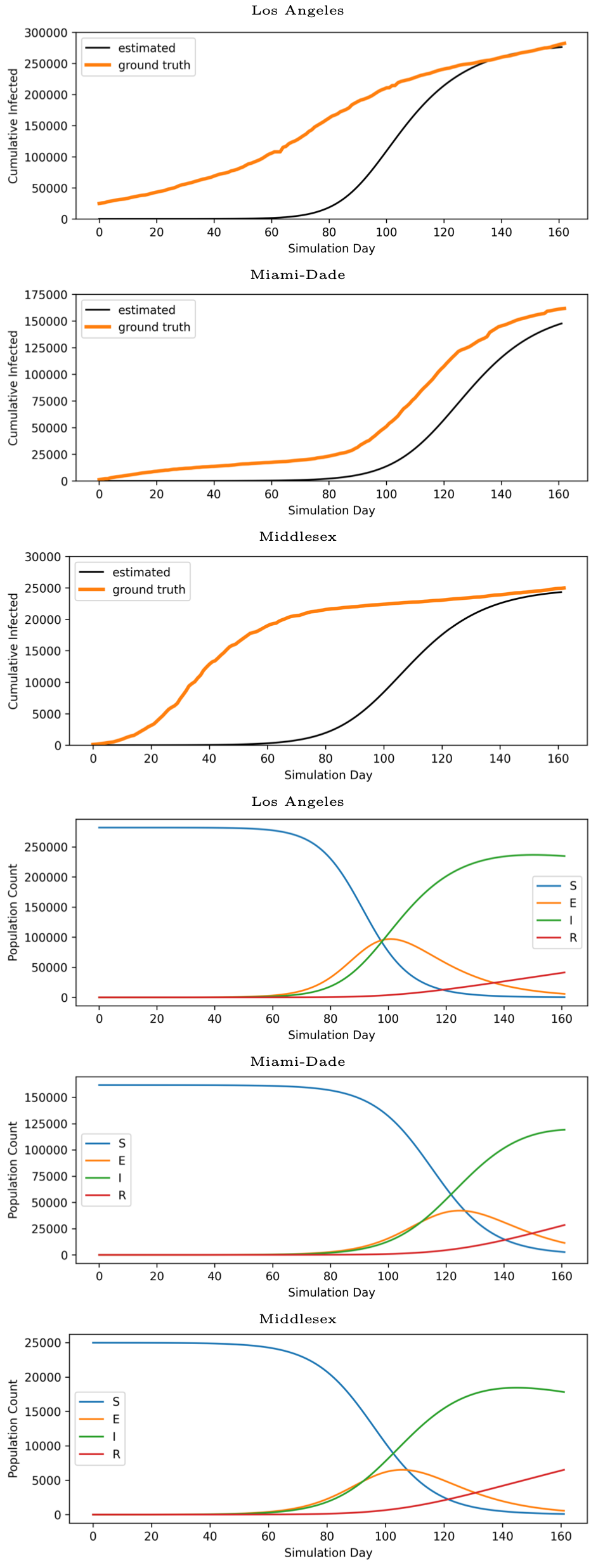

Supplement: S1 Fig — This model is only capable of outputting a disease history corresponding to a single wave of infection. In Miami-Dade, this allows for a reasonable approximation of the regional case counts, whereas for Middlesex and Los Angeles the fit is much worse. (TIFF) [file pcbi.1010591.s004.tiff]
