# Supplementary material for: Probabilistic program inference in network-based epidemiological simulations
Source: PLoS Comput Biol. 2022 Nov 7;18(11):e1010591. doi: 10.1371/journal.pcbi.1010591 (PMC9671460; doi:10.1371/journal.pcbi.1010591)
Supplement: Attachment — Submitted filename: ReviewReport_PCOMPBIOL-D-21-022232.pdf [file pcbi.1010591.s005.pdf]

Review Report on PCOMPBIOL-D-21-02223 "Probabilistic Program Inference in Network-based Epidemiological Simulations"

In response to COVID-19, many research works have been done to study the dynamics of the disease spread. Every week CDC ensembles various groups' predicts of the number of newly reported COVID-19 deaths, and a list of the works can be found at `https://www.cdc.gov/coronavirus/2019-ncov/science/forecasting/forecasting-us.html?CDC_AA_refVal=https%3A%2F%2Fwww.cdc.gov%2Fcoronavirus%2F2019-ncov%2Fcovid-data%2Fforecasting-us.html`.

The authors proposed a Network-SEIR Model in this work, which comprises two components: (i) a network topology model and (ii) an agent-based compartmental model. Then, the authors used probabilistic program inference methods to approximate the distribution over disease transmission parameters. In general, this is an important topic to study, and I feel the article can be improved in the following aspects.

- When incorporating the network into the model, the authors selected CBGs that contribute to the top 10% of mobility data available for each county during February 2020, shortly before widespread quarantines were instituted in the United States. However, it is crucial to notice that the network is also time-varying and highly affected by many other variables, such as the policies. Since the network is an essential part of the proposed model, it would be great if the authors could incorporate a varying network instead of a fixed one.

- It is nice that the authors studied the robustness of the proposed method to the choices of various networks with different numbers of nodes. Based on the simulation results in Table 4, it was evident that the choices of the networks have a significant effect on the performance of the model. Therefore, it would be more helpful if the authors could provide some suggestions on the way of choosing the networks.

- In this study, the authors used the product of independent logistic normal distributions over each disease parameter as the prior distribution. It would be more convincing if the authors could provide more details on how to determine the prior distribution as well as the effect based on different choices.

- As the authors mentioned, there are many issues with the COVID-19 data since it is such a new infectious disease, such as the under and delay reporting. As

a result, most of the existing studies use the prediction accuracy of the death count as a measurement. It would be nice if the authors could report their results based on both the infections and the deaths.

- There are many studies on COVID-19, and an incomplete list can be found from the CDC's website mentioned above. It would be better if the authors could compare their method with these existing methods to highlight the advantages of the proposed method.

- Some minor issues

  - Add "the" before "most" on Line 28 Page 2.
  - There are question marks on Line 653 Page 19.
  - Missing a space after the "Javascript" on Line 692 Page 20.
