# Supplementary material for: Probabilistic program inference in network-based epidemiological simulations
Source: PLoS Comput Biol. 2022 Nov 7;18(11):e1010591. doi: 10.1371/journal.pcbi.1010591 (PMC9671460; doi:10.1371/journal.pcbi.1010591)
Supplement: Attachment — Submitted filename: comments.pdf [file pcbi.1010591.s006.pdf]

In the manuscript titled "Probabilistic Program Inference in Network-based Epidemiological Simulations", the authors conducted a very interesting case study of using variational inference approach to fit an agent-based SARS-CoV-2 transmission model to the observed epidemic curve in three different urban areas in the US, based on real-world contact network data. The authors present a very encouraging exploration of utilizing modern probabilistic programing technics and variational inference approaches to infectious disease modelling problems. For infectious disease modelers, the temptation of fitting more complex and expressive models has long been hindered by the computational challanges of performing traditional inference such as MCMC or likelihood profiling, especially for simulation-based approaches, where the likelihood function of the full model cannot be explicitly written down. Variational inference approaches that have seen rapid progress with application to large scale models in machine learning community provides a promising alternative. And analysis presented in this study could open up further explorations in adopting state-of-the-art numerical methods from machine learning to infectious disease modelling problems. Overall, the manuscript is clearly writing, and the analysis is well conducted. I would recommend the manuscript for publication besides a few comments and suggestions listed below:

- The NSEIR model has a model of contact networks based on Safe-Graph data. During the modelling period of interest, do the authors assume a static network structure over the time? If so, this may not reflect the reality since during the pandemic, different NPIs were implemented/lifted at different time and thus would impact individuals' behaviors and contact patterns throughout the pandemic. The effects of NPIs on transmission is one of the key drivers of the epidemic trajectory. I wonder if the underlying Safe-Graph data have good temporal resolution so that the authors could implement time-varying contact network that may reflects the impact of NPIs over time without explicitly modelling them.
- For inference problem in infectious disease modeling, we not only care about the best estimates on the inferred disease parameters but also their confidence intervals, conditional on the available evidence (observation data). Based on equation (7), variational inference should be able to provide the approximated full joint posterior of the parameters of interest. It's necessary for the authors to present the marginal posterior distribution of all inferred parameters including beta_E, beta_I and rho, rather than only presenting the median estimate of one of the parameters (beta_E) in Figure 6.
- Since the authors only fit the model to incidence time series, it's possible that for Beta_E and Beta_I, there is not enough signal from the data for the model to independently identify them separately, and the two parameters are potentially highly correlated. This is also known as "parameter identifiability issue" (e.g. due to over fitting). I would recommend the authors to check the joint marginal posterior distribution between beta_E and beta_I (for the same time period) to see if beta_E and beta_I are correlated and thus the issue of parameter identifiability exits. Ideally, joint posterior distribution of all parameter pairs (i.e. Beta_E vs Beta_I, Beta_E vs rho, and Beta_I vs rho) need to be checked and presented as part of the inference results.
