# Supplementary material for: Probabilistic program inference in network-based epidemiological simulations
Source: PLoS Comput Biol. 2022 Nov 7;18(11):e1010591. doi: 10.1371/journal.pcbi.1010591 (PMC9671460; doi:10.1371/journal.pcbi.1010591)
Supplement: Attachment — Submitted filename: author_response.pdf [file pcbi.1010591.s007.pdf]

Author Response and Summary of Revisions

We thank the reviewers for their time and attention during this review process and for their detailed and thoughtful comments. Both reviewers overall appear positively predisposed towards this submission and offer a number of concrete suggestions for further improvements.

Reviewer 1 considers disease modeling and associated methods for parameter estimation an important topic of study. The reviewer also appreciates the analysis of the effect of the choice of network. To further improve the manuscript, the reviewer suggests to consider time-dependent networks, provide more detailed motivation for the choice of prior and its effects on posterior inferences, further consider the effect of the network size, report results based on both infection counts and deaths, and to compare and contrast the employed model to other models, such as those for which results are reported on the CDC website. We have incorporated these suggestions where possible and respond to each of these points below.

Reviewer 2 finds this work to be a very interesting case study of the application of variational inference methods for probabilistic programs to parameter estimation in disease models. The reviewer finds the paper clearly written and the analyses well conducted, and recommends publication. Suggestions for further improvement by the reviewer are to consider a model variant with time-dependent network connectivity, report (approximate) posterior marginals, and determine whether the rate parameters β^I and β^E are correlated in the posterior. We have followed up on these points and report on them below.

Time-varying networks. A point raised by both reviewers was that it would be useful to look at a variant of the model in which the network connectivity is time-dependent, since public policy interventions can strongly affect the connectivity. We completely agree with this point, and we did in fact already include some experiments that employ a time-varying contact network (previously Section 5.7 paragraph 3 "Sensitivity to Evolving Network Connectivity Patterns"). However, we understand why the reviewers may have overlooked these results, since they were not emphasized sufficiently. To address this shortcoming we have revised the manuscript to make these results more prominent, (now Section 5.8 "Sensitivity to Time-Varying Network Model").

Additional Changes In addition to changes requested by the reviewers, we have made minor additional changes throughout. All changes are highlighted

in blue in the revised manuscript.

Reviewer 1

Effect of network size on performance. The reviewer indicates that model performance varies with network size and asks for conclusions on how to choose a network based on these observations. We believe this may be a slight misreading of our results. We do observe that inferred parameter values depend on the network size (Table 4 and Figure 13). However, the quality of fit (MDAE; also in Table 4) does not appear to strongly depend on the network size. The dependence of the parameter values is to be expected; we scale the infection data to the downsampled graph before fitting and the infection must therefore spread more slowly from node to node to achieve the same scaled trajectory. However, we do not see evidence that model performance is strongly affected by the network size. We have edited Section 5.7 to clearly highlight this interpretation.

Consequently, the network size and other network modeling choices should be largely dictated by the resolution of available data, downstream modeling needs, and inductive biases designed to increase realism. Generally speaking, one does not need to take parameter fitting into account; our method will adapt parameters to the modeled network. Choosing a larger network size by increasing the number of communities will give a higher resolution simulation, but this comes at a cost of introducing more parameters to be fit and increasing the computational cost of parameter fitting.

For example, we choose our network model from the popular category of mobility networks called spatial meta-population [2, 3, 1]. Our model uses Census Block Groups to define spatial communities (versus counties, or geographic areas around airports used in other meta-population models). In addition, our network model uses a degree-correction procedure that generates heterogeneous mixing patterns within each community, patterns that match other realistic contact networks of similar sizes [4, 5, 6].

We choose the number of communities by analyzing convergence properties of core topological properties like network density and number of triangles. The sizes of each community and the density of contact patterns within and across communities are selected to match census and Safe graph data from the corresponding Census Block Groups.

Choosing the prior distribution. We designed the structure of our prior and variational distribution to be as simple as possible while fulfilling several a priori constraints. In particular, the ρ parameter, describing initial exposure fraction, must be in $[0, 1]$. Likewise, we choose to restrict the parameters β^E and β^I to the range $[0, 1]$ such that the maximal contribution of each node to the exposure pressure and infection pressure of its neighbors is controlled by its edge weight. The logistic normal distribution is a simple choice that satisfies these criteria. In particular a normal distribution would be unbounded

and log-normal distribution would be unbounded above. We have revised the description of the design of the prior in Section 4.3 to reflect this reasoning.

Report results based on both infections and deaths. Thank you for this suggestion. We have added a new experimental section (5.9) to the paper in which we condition our model on both death and infection data. Besides MDAE on infections, we introduce MDAE on deaths as a separate metric to show goodness of fit to both infection and death data. We find that our model is able to find parameters which fit both types of data simultaneously.

Comparing with existing methods. We chose not to compare the state-of-the-art *forecasting* methods listed on the CDCs website. This is because our method does not model the evolving temporal dynamics of the disease. We are able to fit time-varying infectiousness parameters over a past time range based on data, but our model cannot extrapolate into the future except by assuming these parameters stay constant. Thus it is unlikely to be effective for forecasting compared models such as Wu et al. [7]. The goal of our method is to provide a realistic simulator of previous events to allow experimenting with control strategies and counterfactual histories. We have updated the introduction and discussion of baselines in Appendix Section B to clarify this point.

Minor issues. Thank you. We have fixed these.

Reviewer 2

Posterior marginals. Thank you for this suggestion. We have added plots (Figure 6 and Figure 11) illustrating the marginal posterior distribution over β_E, β_I and ρ across all three counties and knots. We note that Figure 7 (old Figure 6) contains a ribbon illustrating the 25 and 75-th percentiles in addition to median.

We would like to note there are some limitations to the computational approach to variational inference in this paper which may impact the accuracy of our posterior uncertainty estimates. A limitation that we already discussed explicitly in the original manuscript is that we use a fully-factorized variational distribution over parameters, which means that the posterior approximation does not capture correlations between variables. The accuracy of our uncertainty estimates may also be affected by the the signal-to-noise ratio of the gradient estimator. See below for further discussion of these points.

Parameter Identifiability. We agree with these observations about parameter identifiability; it is true that different disease dynamics can result in the same observed case counts. The reviewer is also correct that this could result in a correlated and/or possibly even a multi-modal posterior. However, the fact that we employ a factorized variational distribution makes it difficult to

uncover such correlations, particularly given our observations about the noise of the simulator.

However an arguably more relevant limitation, which we feel is worth highlighting more explicitly, is that we treat the simulator as opaque. This means that $q_\phi(\theta) \simeq p(\theta \mid x)$ approximates the posterior marginal, but that $q_\phi(s \mid \theta) = p(s \mid \theta)$ is simply the distribution over trajectories in the simulator given input parameters θ. As we show in Figure 12, this results in a trajectories with a high variance (even when parameters are fixed), which in turn implies that the accuracy of variational inference may be limited by the signal-to-noise ratio of our gradient estimate. Since we initialize the variational distribution to the prior, it is therefore possible that the final variational estimate is not as tightly peaked as the posterior. To emphasize this point more clearly, we have added discussion in the limitations section (page 2) as well as discussion in a new Section 5.10.

We did investigate whether it is possible to use importance sampling (using the variational distribution as a proposal) to characterize correlations in the posterior. However we found that importance sampling estimates are also high variance. The main reason for this is that we employ a *sharply peaked* likelihood function which strongly penalizes trajectories that deviate from the observed case counts. We found this was required to produce realistic case count trajectories, i.e. trajectories for which the mean daily absolute error is low. However a drawback of doing so is that it makes the task of generating samples from the posterior marginal more challenging, as there will always be a small number of samples that dominate other samples in terms of likelihood by orders of magnitude (due to the sharply peaked likelihood function). The net result is that importance weighting strategies to approximately visualize posterior marginals such as $p(\beta_1^E, \beta_1^I|x)$ become extremely sample inefficient. We experimented extensively with other likelihood functions for which learning an ideal proposal / guide is easier and hence quantifying the posterior marginals with importance sampling techniques is feasible. However, we found that employing such a likelihood function resulted in case count trajectories which deviated too much from the observed daily case count.

References

[1] Aniruddha Adiga et al. "Mathematical models for COVID-19 pandemic: a comparative analysis". In: *Journal of the Indian Institute of Science* 100.4 (2020), pp. 793–807.

[2] Matteo Chinazzi et al. "The Effect of Travel Restrictions on the Spread of the 2019 Novel Coronavirus (COVID-19) Outbreak". In: *Science* 368.6489 (2020), pp. 395–400.

[3] Vittoria Colizza and Alessandro Vespignani. "Epidemic modeling in metapopulation systems with heterogeneous coupling pattern: theory and simulations". In: *Journal of Theoretical Biology* 251.3 (Apr. 2008), pp. 450–467. ISSN: 0022-5193.

[4] Nathan Eagle and Alex Pentland. *Reality Mining: Sensing Complex Social Systems*. 2005.

[5] Mathieu Génois and Alain Barrat. *SocioPatterns datasets*. Zenodo, Dec. 2017. DOI: 10.5281/zenodo.2540795. URL: https://doi.org/10.5281/zenodo.2540795.

[6] Marcel Salathé et al. "A High-resolution Human Contact Network for Infectious Disease Transmission". In: *Proceedings of the National Academy of Sciences* 107.51 (2010), pp. 22020–22025.

[7] Dongxia Wu et al. "DeepGLEAM: a hybrid mechanistic and deep learning model for COVID-19 forecasting". In: *arXiv preprint arXiv:2102.06684* (2021).
